# A behavioural study of obedience in health professional students

**DOI:** 10.1007/s10459-021-10085-4

**Published:** 2021-11-22

**Authors:** Efrem Violato, Brian Witschen, Emilio Violato, Sharla King

**Affiliations:** 1 Department of Educational Psychology, Faculty of Education, University of Alberta, Education North, 11210 - 87 Ave, Edmonton, AB T6G 2G5 Canada; 2 School of Health and Life Sciences, Northern Alberta Institute of Technology, Edmonton, Canada; 3 Department of Psychology, Katholieke Universiteit Leuven, Leuven, Belgium

**Keywords:** Compliance, Displacement of responsibility, Moral foundation, Individual differences, Obedience to authority, Positive deviance, Speaking up

## Abstract

Interprofessional Education and Collaborative Practice (IPECP) is a field of study suggested to improve team functioning and patient safety. However, even interprofessional teams are susceptible to group pressures which may inhibit speaking up (positive deviance). Obedience is one group pressure that can inhibit positive deviance leading to negative patient outcomes. To examine the influence of obedience to authority in an interprofessional setting, an experimental simulated clinical scenario was conducted with Respiratory Therapy (RT) (n = 40) and Advanced Care Paramedic (ACP) (n = 20) students. In an airway management scenario, it was necessary for students to challenge an authority, a senior anesthesiologist, to prevent patient harm. In a 2 × 2 design cognitive load and an interventional writing task designed to increase positive deviance were tested. The effect of individual characteristics, including Moral Foundations, and displacement of responsibility were also examined. There was a significant effect for profession and cognitive load: RT students demonstrated lower levels of positive deviance in the low cognitive load scenario than students in other conditions. The writing task did not have a significant effect on RT or ACP students’ behaviour. The influence of Moral Foundations differed from expectations, In Group Loyalty was selected as a negative predictor of positive deviance while Respect for Authority was not. Displacement of responsibility was influential for some participants thought not for all. Other individual variables were identified for further investigation. Observational analysis of the simulation videos was conducted to obtain further insight into student behaviour in a compliance scenario. Individual differences, including experience, should be considered when providing education and training for positive deviance. Simulation provides an ideal setting to use compliance scenarios to train for positive deviance and for experimentation to study interprofessional team behaviour.

**Supplementary Information:**

The online version contains supplementary material available at 10.1007/s10459-021-10085-4.

## Introduction

In the twenty years after the 1999 Institute of Medicine report, *To Err is Human* (Kohn et al., 1999), iatrogenic sources of death continue to be a major challenge globally (Cohen & Patel, 2020). One of the most prominent means to improve patient safety that has emerged is the focused integration of different healthcare professionals into cohesive teams through Interprofessional Education and Collaborative Practice (IPECP) (Cosby, 2017; Frenk et al., 2010; Kohn et al., 1999; World Health Organization, 2010). IPECP has grown over the last two decades (Reeves et al., 2017), and is often presented as a panacea to numerous problems in healthcare. It has been proclaimed Interprofessional Education (IPE) is a great truth awaiting validation (Gilbert, 2013) and that the benefits of Interprofessional Collaboration (IPC) are clearly documented and the need for IPE in undergraduate and graduate education is supported by the literature (Wellmon et al., 2017).

“In practice, however, groups often fail to live up to their potential, largely because of social interactions that may constrain individuals from fully participating in generating ideas and sharing knowledge (Hill, 1982 in Croskerry, Cosby, 2017 pp 213).

At present, the evidence is not so unequivocal. The literature does not demonstrate IPECP is uniformly beneficial, with mixed results for patient outcomes (Didier et al., 2020; Lapkin et al., 2013; McCutcheon et al., 2020; Paradis & Whitehead, 2018; Vuurberg et al., 2019) and with some aspects of IPC being negative (Beran et al., 2014; Kaba, Beran, et al., 2016; Kaba, Wishart, et al., 2016). For instance, conformity to group influences leading to the misreporting of vital signs (Kaba Beran, & White, 2016; Kaba, Wishart, et al., 2016). Concerns for patient safety and a focus on IPECP has led to long existing issues in healthcare education and practice being brought to light, in particular communication in hierarchies and the ability to challenge colleagues when something does not seem right (Green et al., 2017; Pian-Smith et al., 2009). In healthcare, group communication and team interactions have historically not been well investigated (Cosby, 2017). However, a growing body of research reports issues with compliance through hierarchies and professional cultures (Alingh et al., 2019; Holmes et al., 2014; Mak-van der Vossen et al., 2018; Martinez et al., 2017; Pattni et al., 2019; Peadon et al., 2020; Schwappach et al., 2019), conformity and peer pressure (Beran et al., 2013, 2015; Kaba & Beran, 2016; Kaba, et al., 2016a, b) and authority (Bould et al., 2015; Calhoun et al., 2014; Delaloye et al., 2017; Friedman et al., 2015; Kuo et al., 2020; Shanks et al., 2020; Sydor et al., 2013).

While these aspects of group dynamics can be determinantal, the situation is complex as hierarchies, professional cultures, conformity, and obedience to authority can be necessary for learning and professional practice. A detrimental effect of group dynamics occurs through negative compliance: the potential negative consequences that can arise from deference, yielding, or complying with others (Delaloye, 2017) or when a person does not speak up or alter a course of action believed to be inaccurate or unsafe (Green et al., 2017). Negative compliance can function broadly through Groupthink and more specifically through conformity and obedience to authority (Kaba, Beran, et al., 2016; Kaba et al., 2016). In the healthcare literature the behaviours that comprise negative compliance have not typically been termed as conformity and obedience but are discussed generally as ‘barriers to speaking up’. Though the literature on negative compliance is nascent, strong effects have been shown (Pattni et al., 2019; Peadon et al., 2020). As interdependence and teamwork amongst health disciplines increases it is important to study Conformity and Obedience to understand how interprofessional teams can fall short of the ideal and produce negative patient outcomes (Hémon et al., 2020; Kaba et al., 2016; Kaba, et al., 2016). Examining the negative aspects of teamwork, along with the positive outcomes, is prudent for determining how IPECP can exacerbate the issues IPECP has been posited to solve.

Existing research on compliance has largely focused on interactions between physicians and nurses (Blenkinsopp et al., 2019; Pattni et al., 2019; Peadon et al., 2020) leaving other health professions, in particular students in allied health professional programs, as an under researched group (Milligan et al., 2017). Understanding how compliance and difficulties in speaking up affects other essential members of the healthcare team (Milligan et al., 2017; Peadon et al., 2020), such as Respiratory Therapists (RT) and Advanced Care Paramedics (ACP), and students in other allied health professions, is important to fully comprehend team dynamics among all members of interprofessional teams in hospital and prehospital environments. Some studies on teamwork and speaking up have included RT’s (Pattni et al., 2019) though literature on practitioners such as ACP’s is absent (Kilpatrick et al., 2020). It is necessary to develop knowledge about how students outside of medicine and nursing with different roles and positions in the healthcare hierarchy are affected by compliance.

### Compliance

Group dynamics, situations where two or more people interact for a common purpose (Tasca, 2020), have a long history of study in psychology and are ideal for studying compliance in health care teams (Beran, 2015; Kaba et al., 2016; Kaba et al., 2016; Lewin, 1947a, b; Weiss et al., 2014). Compliance is operationalized as “A particular kind of response—acquiescence—to a particular kind of communication—a request. The request may be explicit… or it may be implicit” (Cialdini & Goldstein, 2004). The explicit request can be overt using forceful or nonforceful means, while the implicit can include social forces that are subtle, indirect, and nonconscious (Cialdini & Goldstein, 2004). The forces used can be real or imagined and create internal or external change in a person (Barrett, 2017).

Mitigating negative compliance is difficult as much of human psychology, including obedience and conformity, is generally non-conscious (Cialdini & Goldstein, 2004; Haidt, 2001). Obedience and conformity are ecologically rational social-cognitive heuristics that function well for solving cognitive and social problems when considered against commonly encountered cognitive and environmental constraints (Campitelli & Gobet, 2010; Gigerenzer, 2010; Gigerenzer & Goldstein, 1996). Compliance is a survival mechanism that is very difficult to break from (Friedrich, 1993). Cognitive Load, Individual Characteristics, and displacement of responsibility, among other variables, function as constraints that make breaking from compliance difficult.

#### Cognitive load

Cognitive Load Theory (CLT), how memory and learning is influenced by different stimuli, is a leading model in educational psychology, generalizable across domains (Szulewski et al., 2021), yet in compliance research is a long-standing under-investigated variable (Baker, 2019; Baron et al., 1996). Cognitive load may be particularly important for compliance scenarios in healthcare where numerous external stimuli can increase cognitive load (Sewell et al., 2020). If practitioners are experiencing high levels of cognitive load, it may be difficult to access adaptive structures or tools for speaking up as automatic and effortful modes of processing are interrupted and situational awareness is reduced (Elfering et al., 2015; Grzyb et al., 2018). Delaloye (2017) found deferring to authority allowed healthcare professionals to manage cognitive load and focus on a single task.

#### Individual characteristics

The influence of individual differences on speaking up has been examined to varying degrees with indeterminant findings related to personality traits, confidence, self-efficacy, and profession (Barzallo Salazar et al., 2014; Daly Guris et al., 2019; Kuo, et al., 2020; Oner, et al., 2018; Roussin et al., 2018; Sydor et al., 2013). In general, sex differences are not influential in obedience to authority (Blass, 1999); though contexts may exist where sex differences are important (Pattni et al., 2017; Roussin et al., 2018).

A previously unexamined, though potentially informative theory for understanding individual differences is Moral Foundations Theory (MFT). MFT is a predominant theory in moral psychology with strong evidentiary support. MFT suggests that rather than engaging in careful moral reasoning humans are moral intuitionist (Graham et al., 2018; Haidt, 2001; Mikhail, 2021). The moral intuitionist perspective is that moral reasoning is post-hoc and follows intuitive moral judgment, rather than moral reasoning producing moral judgment through a process of ratiocination. Moral judgments are influenced by individual dispositions and cultural variability on five foundational moral intuitions: Harm/Care, Fairness/Proportionality, Purity Sanctity, Ingroup Loyalty, and Respect for Authority (Haidt, 2012). Moral intuitions are quick, effortless, and automatic thoughts, otherwise known as heuristics (Gigerenzer, 2010). Respect for Authority in particular is an adaptive heuristic for the complex and dynamic social systems that govern social functioning and can have a strong influence in affecting obedience to authority (Graham et al., 2018). MFT is useful for understanding compliance that can produce medical errors by providing a basis for examining how cognition unbeknownst to the human actor that produces a disposition towards obedience may influence behaviour.

#### Displacement of responsibility

People may obscure or minimize an agentive role in harm by viewing their actions as stemming from the dictates of an authority, this is motivated reasoning known as displacement of responsibility (Bandura, 2002). Displacement of responsibility has been consistently identified as one of the most important variables for creating obedience to authority (Bandura, 1999; Richardot, 2014). The effect also appears to be present in the context of healthcare. In compliance scenarios the displacement of responsibility has been identified as preventing action by causing people to feel less responsible and become “agents of their leader.” (Bould et al., 2015; Friedman et al., 2015).

#### Mitigating negative compliance

To mitigate negative compliance caused by obedience, it is necessary to enact Positive Deviance (PD). Positive Deviance is effectively taking action to prevent harm and negative consequences to a patient and counter behaviour that erodes professional values or creates negative outcomes (Blanton & Christie, 2003; Holmes et al., 2014). The action is “deviant” because it is taken regardless of whether others take the action or if the action is socially supported or reinforced (Blanton & Christie, 2003; Holmes et al., 2014). Positive Deviance can occur through speaking up or challenging authority (Pattni et al., 2019) or may include other actions such as adhering to procedures when others do not. Promoting PD involves helping students or health professionals resist pressure to enact unsafe or unprofessional practices (Holmes et al., 2014). To date the interventions developed to promote PD, including speaking up, have proven inconsistent and variable (O’Donovan & McAuliffe, 2020).

Ariely (2008) popularized the idea that though people are not rational they are “predictably irrational”. People’s biases, misjudgments, heuristics, causational inferences, and cognitive illusions function in similar and highly predictable ways. If a person’s behaviour is predictable then it can be targeted, and the locus of change and intervention should be placed on the individual rather than primarily on broad social and professional structures (Bainbridge & Regehr, 2015; Holmes et al., 2014). In other words, effectively promoting PD might best occur through targeting individual’s behaviour.

To modify individual behaviour change should focus on a lower level of cognition, the person’s self-concept. Self-concept change can influence a person’s beliefs, attitudes, perceived control, and subsequent behaviour (Hogarth, 2001). Self-concept change can be enacted through the principle of consistency (Cialdini, 2006). Self-concept is maintained by consistent behaviour and will modify a standard intiutive response (Cialdini, 2006). Behaviour not aligned with the self-concept will create cognitive dissonance (Festinger, 1957; Festinger & Carlsmith, 1959; Mcgrath, 2017) and threaten self-esteem. The inherent desire and need to protect self-esteem can be resolved by acting to protect the self-concept (Greenwald et al., 1988; Pyszczynski et al., 2004). A person may enact PD by speaking up or taking action to resolve a situation if their self-concept is modified so that being obedient when they perceive something is wrong becomes a major threat to their self-esteem. In other words, engaging in PD produces benefit for the person through reducing dissonance by aligning their behaviour with the self-concept of being someone who speaks up. Consistency is created, and self-esteem and self-concept are maintained. Undesirable obedience is altered through the positive application of consistency of thought and behaviour, a guiding principle of human cognition (Cialdini & Goldstein, 2004) and one of the most basic social heuristics (Bocchiaro & Zimbardo, 2017).

A simple method to achieve self-concept change is through a writing task. If the initial action for a change was active, effortful, and viewed as internally motivated, the creation of a need for consistency will be most effective (Cialdini & Goldstein, 2004; Cialdini & Trost, 1998). To create the internal motivation and change in self-concept a writing task can elicit effortful activity. Expressing a certain position formally through writing or speech, particularly if the position is made public, will cause a need for behaviour or thought that is consistent with the expressed opinion (Cialdini, 2006). Defending or espousing a certain position, whether the position was taken voluntarily or involuntarily, can cause a person’s beliefs and attitudes to shift towards the position taken (Gastil et al., 2008; Schug, 1954; Wojcieszak, 2011). With a shift in belief and attitude comes a change in self-concept to the extent that to be consistent and maintain self-concept the person will have to modify their behaviour. With a writing task, the self-concept change would occur by having students write about the possible harm that can occur to a patient if the student follows incorrect instructions or fails to speak up when observing an action they believe to be inaccurate or harmful. Students would also write about what they would do to ensure the patient was not harmed.

#### Purpose of the study

The present study will focus specifically on how the social-cognitive heuristic of obedience to authority creates negative compliance in the context of an interprofessional team. This study will examine three facilitating variables for obedience in the context of an interprofessional team: cognitive load, individual characteristics including the disposition to respect for authority, and displacement of responsibility. An interventional writing task intended to improve PD though self-concept change will also be tested. Understanding the variables that create compliance in interprofessional teams can improve understanding of how the environment and individual interact and how efficient evidence-based change can be instituted.

### Research questions

To fulfill the study purposes two sets of research questions were developed:

Primary

1. Will Respiratory Therapy and Advanced Care Paramedic students demonstrate Positive Deviance in a simulated clinical scenario?
2. Will high cognitive load decrease the rate at which students demonstrate Positive Deviance, conversely will high cognitive load increase Obedience?
3. Can a brief writing task increase the rate at which participants demonstrate Positive Deviance, conversely will the intervention decrease obedience?

Secondary

1. Will any individual characteristics be predictive of Positive Deviance?
2. Will Displacement of Responsibility inhibit Positive Deviance?
3. What insights can be obtained regarding Obedience and Positive Deviance through observation of the simulation scenarios designed to enact obedience?

## Methods

### Design

The study used a 2 × 2 factorial experimental design. The manipulated variables were Cognitive Load (High/Low) and a Writing Task (Intervention/Control). The study was conducted using a simulated airway management scenario where the participant would be assisting a senior physician with a difficult intubation. The situation would become dangerous for the patient as the physician persisted with obtaining the airway. Rates of PD were measured through direct observation of the simulation. To elicit authentic behaviour during the simulation, deception was used. Participants were told the research was part of a personality study intended to develop individualized learning for simulation training. Participants were debriefed after the simulation and the full nature of the study was revealed including the purpose of the writing task and the reason for using deception. At the end of the debriefing participants were checked for discomfort and consent was reaffirmed. The study was conducted during the 2019 Winter Semester and was approved by the Northern Alberta Institute of Technology Research Ethics Office and the University of Alberta Research Ethics Board 2.

An airway management scenario was selected as it has been previously shown to be practical for examining PD (Pattni et al., 2017) and airway management is an important aspect of patient safety. Failure to intubate and hypoxemia is an important factor for error in care and a major cause of morbidity and mortality (Griesdale et al., 2012; Langeron et al., 2018).

#### Participants

Participants were recruited from the second-year RT cohort, 40 students, and first-year ACP cohort, 20 students, at the Northern Alberta Institute of Technology. Both groups are experienced with simulation training and have uniform knowledge in performing airway management tasks. Approximately two weeks prior to the first stage of the study, participants had performed instructor-led lab scenarios requiring them to speak up and advocate for patient safety during a critical incident. Participants were recruited during class time to participate in the study and time normally allotted for simulation training was used for the study. Students were informed that participation was voluntary and choosing not to participate would have no influence on their grades or academic standing.

#### Materials

*Writing Task* The interventional condition for the writing task was designed as described in the introduction to cause a person to see themselves as someone who engages in PD. A neutral writing task was developed as a control. Participants were asked to not discuss the writing task with their peers. Three different neutral writing tasks were used in the case that participants did discuss the writing task it would not be obviously apparent there was an intervention and control writing task. The control writing task included either writing about a favourite summer vacation, a favourite place to study, or a favourite past time outside of school. The intervention and control writing tasks were ostensibly for the purpose of personality assessment (Küfner et al., 2010) as a part of the assessment of personality for individualized simulation learning.

*Individual Measures* the Moral Foundations Questionnaire (MFQ), specifically the Respect for Authority subscale, was used to measure individual’s disposition to obedience. The MFQ has demonstrated good validity evidence and at present is the best scale for assessing compliance to authority (Doğruyol et al., 2019; Graham et al., 2018; Matsuo et al., 2019; Nilsson & Erlandsson, 2015). The remainder of the survey collected data on age, sex, GPA, experience and confidence with airway management, clinical and simulation experience, and post-secondary education.

*Cognitive Load* High Cognitive Load (HCL) was created by having a Standardized Patient (SP) play a distressed family member of the patient. To increase cognitive load for the participant the actor was instructed to appear distraught and emotional, question the participant and the doctor, demonstrate concern for the patient’s well-being and speak to the patient. All family members were females between the ages of 40–60 and were instructed to indicate they were the patient’s sister.

*Doctor* The doctors were played by SPs. All actors were Caucasian males, between 40 and 60 years old, approximately 5′8 to 6 feet tall, and were selected to have an authoritative appearance. Prior to the simulation each actor was trained how to perform an intubation and given as many practice attempts as needed to feel comfortable. A full walk through of the simulation was done with the doctors and family members. The doctor wore an earpiece to receive instruction from the facilitator.

*Patient* The patient was a CAE Healthcare iStan Mannequin (CAE Healthcare, 2017). All participants were familiar with and had practiced on this model of mannequin.

#### Outcome measure

Positive Deviance was defined as the participant making a direct or explicit challenge to the doctor. For a challenge or speaking up to be an effectual intervention it is necessary to be direct or explicit (Garden & Weller, 2017). For example, a direct challenge could include a statement that what was occurring was unsafe, that the doctor needed to stop, that the participant was going to stop the doctor, that harm was being done to the patient, or making a statement about changing the course of action in an assertive tone. The potential approaches to PD, or speaking up, are diverse (Okuyama et al., 2014; Omura et al., 2017) including the two-challenge rule and using an advocacy-inquiry approach (Pian-Smith et al., 2009). As there is no uniformly accepted method for engaging in PD there were no specific phrases alone that were considered to constitute PD, however, the operationalized definition for the study aligns with the final two levels of the Modified Advocacy-Inquiry Score (mAIS) (Sydor et al., 2013). The mAIS has been used to score challenges in a continuous manner (Delaloye et al., 2017; Friedman et al., 2015; Pattni et al., 2017; Pian-Smith et al., 2009; Sydor et al., 2013). The lower levels of the mAIS constitute questions or suggestions. In the present study PD was measured as a binary action, yes or no, for this study a lower-level action was not considered PD. Questions or suggestions directed towards authority or over hierarchical gradients are easier to make as well as dismiss (Islam & Zyphur, 2005; Richardot, 2014). While a direct challenge is more difficult to enact, it removes ambiguity and is more effective in eliciting change (Bandura, 1999).

#### Procedures

**Fig. 1:**
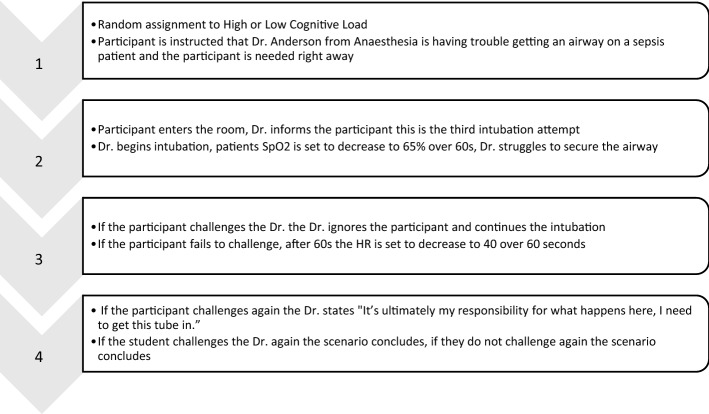
Simulation scenario flow for the experiment

The study was conducted in two stages. Stage One: one week prior to the simulation participants were provided with a link to the consent form, study information, demographic questionnaire, Writing Task, and MFQ, hosted on Qualtrics (Qualtrics, Provo Utah). Participants were given class time to complete the materials. The Writing Task was framed as a personality assessment to understand how different personality types learn in simulation. Participants were randomly assigned to receive either the intervention or a neutral condition. The intervention condition involved writing about how medical errors could occur due to obedience and what action the person would take to prevent such an error. The neutral condition involved writing about either a vacation, studying, or favourite past-time activity. Three neutral conditions were used so that in the case participants discussed the writing task prior to the simulation it would not be apparent there were two conditions, and the true nature of the study would be realized (see Supplemental Material for further detail). Stage Two: one week after Stage One participants completed the simulated clinical scenario. Participants were seconded in a waiting room and brought into the simulation center individually. After completing the simulation, participants were debriefed in a separate room and sent out through an alternate exit so that they would not encounter participants who had not yet completed the simulation. Four simulations ran concurrently. All simulations and debriefings were audio and video recorded for analysis. The scenario flow is shown in Fig. 1 (for full procedures and description of the simulation please see the Supplemental Material).

The deterioration in vital signs is similar to procedures used by Pattni et al. (2017), however, to maintain an open environment there were no specific points where a challenge and reply was expected, thus the participant was able to challenge the doctor at any time during the simulation. The actor playing the physician was instructed to ignore or brush off questions, offers for help, or suggestions. The actor wore an ear-piece and was instructed by facilitators who were program instructors experienced with simulation, airway management, and the need for PD, as to when to use the responsibility phrase: “It’s ultimately my responsibility for what happens here, I need to get this tube in.” The actor would be persistent in trying to intubate the mannequin regardless of the participants behaviour. A soft time limit of two minutes was placed on the simulation. The facilitator was given discretion to allow the simulation to run longer if it appeared the participant would make a challenge or if there was an interesting interaction occurring.

#### Analysis

*Primary Analysis* As there were two categorical predictors and a categorical dependent variable (PD or no PD) log linear analysis was used. Educational Program was included in the analysis as a possible confound. Odds-ratio were used to determine the effect sizes for the two independent variables (IV). A sample size of 60 is adequate for this form of analysis (Stelzl, 2000), post-hoc calculations conducted using GPower (Faul et al., 2007) indicate a power level of 0.87. Primary analysis was conducted in jamovi, a point and click interface for R (jamovi, 2020).

*Secondary Analysis* The data mining/machine learning techniques of Elastic-Net regression (James et al., 2013; Zou & Hastie, 2005), was used to train a model to determine predictors of PD. Elastic-Net regression is ideal for situations where the number of predictors is high relative to N and is advantageous over other variable selection methods (Hong et al., 2020; James et al., 2013). Analysis was conducted using the caret package (Kuhn, 2019) and glmnet package (Friedman et al., 2010) in R (R Core Team, 2019). Two models were trained with different resampling methods, one using fivefold Cross-Validation (CV) with 5 repeats and one using Bootstrapping. A tuning grid was set for alpha from 0–1 and lambda from 0.0001–1 with a search length of 100.

*Observational Analysis* Videos were analyzed using a semi-structured observational approach by two observers. Observers conducted the analysis independently and were blind to the randomization of the writing task. Some behaviours were coded to obtain quantitative measures, such as the number of questions or suggestions and the number of times participants read the blood oxygen saturation. A naturalistic approach was taken for observing any distinct cases, behaviours, or outcomes from the simulations. After independent analysis, the observers compared results for convergence.

#### Data preprocessing

Data were checked for careless responding. There were no inordinately fast completion times for Stage One. All participants completed the writing task with good detail.

Two deception checks were used to determine if participants suspected the true purpose of the study. First, at Stage Two, prior to the simulation participants were asked to identify any familiar names from a list. The list included several famous psychologists including Milgram, Asch, and Zimbardo, known for their work on compliance. Second, after the simulation, during debriefing, participants were asked if they had any suspicion about the true nature of the study. Two participants recognized the name Milgram, one participant indicated they had no suspicion about the true nature of the study and the other indicated some suspicion based on the writing task. Both participants engaged in PD, however, they were not exceptionally fast in their time to PD and didn’t demonstrate any behaviour exceptional from the other students, the participants data was retained. One participant, a Paramedic, indicated they had a degree in psychology and had guessed the purpose of the study and expected the simulation to be about challenging authority. This participant’s data were dropped from the study.

Assumptions of log-linear analysis were assessed prior to analysis. The data came from a random sample of a multinomial and mutually exclusive distribution with all observations being independent (Howell, 2010). The sample size was adequate for the number of variables being assessed, 15 per cell. The expected cell frequencies were also adequate, all cells > 5 (Howell, 2010). Videos were coded for PD by two independent raters. Coders also recorded if PD was engaged in after the physician indicated it was his responsibility for what happened, the number of questions or suggestions a participant made, and the number of times the participant read the Sp02. For PD initial agreement between the coders was 51/59 (86%), Kappa = 0.67, Rater bias ratio = 0.44 χ2 = 0.11, *p* = 0.739. An iterative process of re-coding and discussion was engaged after which there was 100% agreement.

## Results

### Demographics

There was a final total of 19 participants from the ACP program and 40 from the RT program with 28 Females (2 ACP, 26 RT) and 31 Males (17 ACP, 14 RT). Over half of the sample identified as being of Caucasian/European descent 37 (63%), 9 (15%) as Chinese, 5 (8%) as South East Asian, 3 (5%) as an ethnicity not identified on the survey, 2 (3%) of Indian descent, and one each (2%) of Aboriginal, Middle East, and African descent.

ACP students had more clinical experience than RT’s, while RT’s had more experience with airway management. Both groups were comparable on self-rated experience with airway management and confidence with airway management (Table 1). For sample scores on the MFQ see Table 2.

**Table 1:** Demographic characteristics of the sample

		Age	GPA	Yrs of post secondary Ed	Hrs Trained in Simulation	Weeks of clinical experience	Hrs of Exp with airway management	Experience with AW management*	Confidence with AW management^a^
ACP	Mean (SD)	28.2 (5.56)	3.90 (.17)	3.47 (1.65)	45.2 (41.3)	37.6 (31.8)	30.3 (13.4)	3.26 (.73)	3.37 (.68)
	Median [Range]	27 [21–45]	4 [3.44–4.00]	3 [1–6]	25 [3–100]	31 [1–100]	30 [9–60]	3 [2–4]	3 [2–4]
RT	Mean (SD)	24.5 (5.97)	3.64 (.43)	4.13 [2.09]	94.3 (112)	5.95 (16.4)	70.4 (27.3)	3.23 (.77)	3.15 (.74)
	Median [Range]	23 [19–45]	3.8 [2.50–4.00]	4 [1–9]	50 [0–350]	2 [1–100]	77 [23–100]	3 [1–4]	3 [1–4]
Overall	Mean (SD)	25.7 (6.04)	3.72 (.38)	3.92 (1.99)	78.2 (97.4)	15.9 (26.7)	57.5 (30.02)	3.24 (.75)	3.22 (.72)
	Median [Range]	24.0 [19–45]	3.9 [2.05–4.00]	3.00 [1–9]	50 [0–350]	2.00 [1–100]	50 .00 [9.0–100.0]	3 [1–4]	3 [1–4]

^*^ Rated on a 1–5 Likert scale: 1—Not at all Experienced to 5—Very Experienced

^a^ Rated on a 1–5 Likert scale: 1—Not at all Confident to 5—Very Confident

**Table 2:** Sample scores on the Moral Foundations Questionnaire

	Harm/Care	Fairness/ proportionality	In group loyalty	Respect for authority	Purity/ sanctity	Overall
Mean (SD)	22.6 (4.36)	21.1 (3.81)	17.5 (4.51)	17.3 (4.24)	13.2 (5.87)	18.3 (3.27)
Median [Range]	23 [11–30]	22 [11–28]	18 [8–16]	17 [8–27]	11 [3–26]	19 [11–26]

### Primary analysis

Overall, 38 (64.4%) participants engaged in PD, while 21 (35.6%) did not engage in PD (Table 3). A hierarchical four-way log-linear analysis was conducted to examine the effect of Cognitive Load and the Writing Task on PD, with Program as a confound. A significant overall model was identified, χ^2^ (15) = 33.9, *p* = 0.004, *R*^*2*^_*CS*_ = 0.88. There were no significant effects that included the Writing Task. A significant two-way interaction of Cognitive Load x PD was found χ^2^ (1) = 11.97, *p* = 0.005, *z* = −2.81, along with a significant two-way interaction of Program x PD χ^2^ (1) = 5.19, *p* = 0.023, *z* = −2.03. The three-way interaction for Cognitive Load x Program x PD was non-significant χ^2^ (1) = 2.84, *p* = 0.09; *z* = −2.04.

**Table 3:** Rates of positive deviance by conditions

		Engaged in positive deviance			
Condition	Program	Yes		No	
HCL	ACP	8	25	1	4
	RT	17		3	
LCL	ACP	7	13	3	17
	RT	6		14	
		38		21	

To determine the specific effect of Cognitive Load and Program, follow up Chi Square tests were performed. No significant difference in PD was found for ACP students on Cognitive Load, χ^2^ (1) = 1.02, *p* < 0.31; Odds Ratio(95%CI) = 0.29(0.02-0.35), ϕ = 0.23. A significant difference of Cognitive Load was found for RT students with less PD in the LCL scenario, χ^2^ (1) = 12.38, *p* < 0.001; Odds Ratio(95%CI) = 0.08(0.02-0.36), ϕ = 0.57.

The results indicate that the Writing Task had no influence on PD while RT students in the LCL condition were less likely to engage in PD than in other conditions. In the HCL condition students were equally likely to engage in PD.

### Secondary analysis

#### Predictors

The Elastic-Net regression identified several variables predictive of the likelihood of engaging in PD. The CV and Bootstrapping models were generally comparable though CV produced a sparser model with a higher classification Accuracy (SD), 74% (12%), and Kappa (SD), 0.38(0.29), than Bootstrapping 70% (12%) and 0.28(0.27). Results of the CV model are shown in Table 4.

**Table 4:** Results of elastic net regression with fivefold cross validation^*^

Variables	β
Age	− .004
East Indian Ethnicity	.81
Middle Eastern Ethnicity	− 1.25
South East Asian Ethnicity	− 2.32
Confidence in AW Management	− .84
Hours Experience in AW Management	− .02
Harm/Care	.07
Fairness/Proportionality	− .05
In Group Loyalty	− .14

^*^ α = .80 λ = .04

#### Displacement of responsibility

Twenty-one participants did not successfully challenge after the responsibility phrase was used. In several cases, the actor used the phrase on the first challenge or towards a question or suggestion. Though the phrase was not consistently applied, the 21 participants that did not successfully challenge all heard the phrase. Twenty-eight participants that successfully challenged did so after the doctor used the responsibility phrase. Ten participants challenged the doctor in a manner that constituted PD before the doctor was able to use the responsibility phrase. These participants’ challenges occurred very quickly and directly either not giving the doctor the opportunity to use the phrase or continuing the challenge while the phrase was being uttered.

### Observational

Six primary insights were obtained through analysis of the video data: 1) Types of Behaviour, 2) Avoiding Conflict: Making Suggestions and Asking Questions, 3) Displacement of Responsibility, 4) Positive Deviance, 5) ACP and RT Differences, 6) Physical Behaviour & Reactions. These insights inform findings from the primary and secondary analysis and provide further understanding of compliance. The mean time (SD), median (Interquartile Range[Range]) in seconds for the simulation was 93(34), 86.5(69–114[35–200]) s. For successful PD the mean time was 77(23), 75.5(59–89[35–136]) s, for unsuccessful PD the mean time was 123(31), 118(102–143[73–200]) s.

#### Types of Behaviour

Four general types of behaviour were identified across the simulations.

Direct action—participants enter the simulation and immediately attempt to ascertain what the problem was and quickly realize the doctor was struggling and the patient’s condition was deteriorating. The participant would immediately start questioning the doctor’s actions or suggest a different course of action. When the participant challenged the doctor, and the challenge was rejected the participant would persist in engaging in PD.

Delayed action/Realization—participants would enter the simulation and ask what they could do or how they could help but would not do so with urgency or not immediately go to the bedside. The participant might engage in non-task relevant actions. Once the participant realized what was occurring, they would directly engage the doctor. Participants would be persistent in their questions and suggestions though not all would move to PD. Action might be spurred by the family member’s distress.

Inaction—participants initial behaviour was similar to Delayed action/Realization. When the participant realized the doctor was struggling and the patient was deteriorating they would not respond with urgency or if their questions or suggestion were rebuked would draw back. The participant might continue to ask questions or make suggestions in a non-forceful manner but would not attempt to change any course of action, e.g.:

"Sats are at 70, how are you doing? [Doctor: I’m fine. Long pause] how are you doing? [Doctor ignores participant, participant is standing by the side of the bed away from the Dr with their arm resting on bedrail] did you want me to start bagging? [Doctor: No] ok [long pause] heart rate is below 40 and there is no respiratory rate [Doctor ignores participant, participant lowers bed rail and moves further back, participant does not move until the simulation is ended].

Participant #91928 RT Student

Frustrated Inaction—participants initially behaved like the Direct action or the Delayed action/Realization participants. When the participants initial questions, suggestions, or challenge were rebuked the participant would continue with questions or suggestions, however, would not make a direct challenge. As the doctor continued to ignore questions or suggestions the participants would become visually and audibly frustrated. The participant would try to challenge the doctor but would not move beyond suggestions or questions e.g.:

"If you can’t get this next one here I have qualification for intubation [Doctor: responsibility phrase] ok, yah, I, I understand sir [participant moves around the bed. Family member to student: do you know what to do?] yah I do [sharp and frustrated voice, does not challenge the doctor further].

Participant #10000 ACP Student

#### Avoiding conflict: making suggestions and asking questions

All participants realized what was occurring was incorrect and that the patient was in danger, yet many participants’ behaviour was characterized by a desire to avoid conflict. The desire to avoid conflict was apparent in how participants approached the doctor. Participants would ask questions or make suggestions to the doctor, however, they would not make a direct challenge. The questioning and suggesting would carry on and the frequency of the questions and suggestion would increase or the tone of participants voices would change yet participants struggled to move to a direct challenge. Participants questions and suggestions were characterized by words like “should”, “would” “could”, and “probably”, “maybe”, and “I think”; low mAIS statements.

Almost all participants read the blood oxygen saturation (sats) and heart rate out loud. Participants that engaged in PD averaged 3 suggestions or questions and 3 sats readings. Participants that did not engage in PD averaged 4 suggestions or questions 5 sats readings. Several participants almost exclusively read the sats to the exclusion of any other behaviour.

Interestingly there were cases in the first simulations where due to the SP’s uncertainty the doctor was very obviously performing the intubation incorrectly. In some of these cases participants would point out what the doctor was doing wrong yet would not directly challenge:

[Participant makes a few hesitant moves forward while holding bagger] "can I… [trails off], the tube looks like it’s the wrong way"

Participant #88821 RT Student

"Shouldn’t the laryngoscope go down her throat?"

Participant #10001 RT Student

Examples of questions or suggestions used by participants:

[Participant standing back from the bed with hands clasped in front of their body] "Her oxygen sats are at 74% [Doctor: we’re good] I think that’s a little low"

Participant #60698 ACP Student

[Participant picks up bagger and stands beside the doctor] "umm, I’m thinking we should hyper-oxygenate, just get her back up real quickly [Doctor: it’s fine] are you sure… [participant trails off], do you need the bed higher or anything".

Participant #54578 RT student

#### Displacement of responsibility

Some participants were strongly influenced by the doctor’s responsibility phrase. Most participants would continue with questions or suggestions after the doctor made the statement, however, some participants would almost completely disengage from any action, questioning, or suggestions.

"The sats are 60 and they’re dropping, can we just, can we [Doctor: responsibility phrase] yah ok" [spoken very softly and participant backs away from the bedside]

Participant #10001 RT student

"We need to bag her up [Doctor: responsibility phrase] ok [participant backs away, moves forwards and back several times with hands crossed in front of body becoming visibly uncomfortable].

Participant #48456 RT Student

#### Positive deviance

The shift from questioning, suggesting, and offering help to PD was often distinct and included a change in the participants tone of voice. The participants would become distinctively more assertive and phrases, though structured as questions or suggestions, would become statements prior to the actual PD e.g. *“ok if they’re down to 70 we should pre-oxygenate”, “can we please bag?”*. Not all participants engaged PD assertively, some participants maintained an even tone of voice while making it clear that the doctor needed to stop. Others maintained a degree of deference while attempting to engage in PD e.g.

"Can I kind of stop the intubation here sir I honestly think it would be in the best interest of the patient, I hate to be pushy"

Participant #83695 RT Student.

Several participants made physical contact with the doctor after being ignored. Some participants gently placed their hand on the doctor’s shoulder or arm while others would attempt to move the doctor’s hands to remove the laryngoscope or place the bagger on the patient. No participants were aggressive when making contact and only did so when the sats were very low, the doctor ignored questions or suggestions, and after the doctor rejected a challenge with the responsibility phrase.

No common or standard phrase was used by participants, however, all PD phrases included some aspect of the definition of PD as operationalized in the methods. One participant used an advocacy-inquiry approach:

[participant gets to the doctors level and speaks in an even tone] "I know you've tried to intubate twice but what have you done differently the second or the third time?"

Participant #61285 ACP Student

Some participants used the doctor’s responsibility phrase to engage PD:

"I would prefer that we pre-oxygenate sir [Doctor: responsibility phrase] we’re all responsible for the patient’s condition.”

Participant #47471 ACP Student

“Ok doctor I think it is best for the patient that we bag the patient up before we try the next attempt [participant picks up bagger, Doctor: responsibility phrase] ok but it’s my responsibility for the patient as well”

Participant #11411 RT Student

Examples of PD statements:

"Sir, Dr. Anderson, uh uh, just for the patient’s safety I think we’ll have to stop you here"

Participant #63060 RT Student

"I’m going to start bagging ok" [moves in past the doctor to bag]

Participant #10372 RT Student

"Ok doctor I think we’ll have to call someone else to help"

Participant #36873 RT Student

#### ACP and RT differences

There were general differences in behaviour between the ACP and RT students. ACP students tended to be much more direct and assertive than the RT students while RT students appeared to have a greater desire to avoid conflict. The preponderance of participants that engaged in physical action were ACP students. The tone of ACP students generally, though not exclusively, was much blunter than RT students.

When ACP students entered the simulation they tended to go directly to the beside and be in close proximity to the doctor. RT students tended to stand back from the bed, some at a substantial distance, and wait for direction from the doctor, not moving closer until the patient’s sats had dropped. Besides physical positioning RT students were generally more hesitant and less confident.:

[Family member to participant: Do you know what you’re doing?] "umm the doctor does"

Participant #80031 RT Student.

#### Physical behaviour & reactions

When a participant’s suggestions or questions were rejected or ignored by the doctor, or their challenges were dismissed many participants displayed physical frustration or agitation. Physical displays included hesitant moves toward the doctor or the patient before backing off, shifting their weight from foot to foot or forward and backward, grimacing or displaying a puzzled or confused look, and taking deep inhalations with forceful exhalations. The frustration or exasperation was also often apparent in participants voices, including when answering questions from the family member.

When the simulation ended many participants laughed in a relieved manner, made a joke to the doctor, or displayed awkwardness about what to do until the facilitator told them they could leave the simulation room. At the end of the simulation a few participants made statements such as, “*That is tough, very hard!*” or “*Oh that was it*” having realized it was necessary to directly challenge the doctor to end the simulation.

## Discussion

The results of the experimental simulation indicate that cognitive load, is a factor in PD, however, the direction of the influence was counter to the expected outcome. RT students in the LCL scenario were less likely to engage in PD. Program was also an important variable for PD with RT students in the LCL condition engaging in PD at a lower rate than ACP students and those in the HCL condition. The Writing Task did not demonstrate any influence on the rates of PD. Several variables were identified as predictors of PD including ethnicity and confidence in airway management. The observational data supported the findings in the primary and secondary analysis as well as provided insights to PD and potential future questions for research.

### Primary analysis

Positive Deviance and obedience were demonstrated by ACP and RT students with differences between the groups, particularly the frequency of PD. ACP students tended to be older than RT students and had previous clinical experience as Primary Care Paramedics. RT students had more simulation training and more hours of experience with airway management, however, the “real-world” experience and maturity of the ACP students, including interpersonal experience, is likely an important factor in the differences between programs. ACP students may have previously encountered a situation where a patient was at risk due to a colleague’s behaviour, leading to more confidence in acting. Though both groups were comparable in confidence in airway management, general self-confidence and task related self-efficacy (Daly Guris et al., 2019; Roussin et al., 2018) may be more important for PD than confidence in a specific task.

Sex was not a predictor of PD though it is possible the disparity in sex distribution between programs may account for some differences in rates of PD. Males tend to be more aggressive and assertive and have less emotional valence for negative interpersonal interactions (Del Giudice, 2009; Fino et al., 2019), while females are more concerned with inclusion and cohesive group functioning (Lönnqvist et al., 2014).

Different aspects of the professions may appeal to personality differences that exist between people who choose to enter a certain profession (Tesi et al., 2020) and subsequently influence behaviour in compliance situations. Some differences between ACP and RT students have been identified, such as RT students ranking higher on the moral foundations of Harm/Care, Fairness/Proportionality, and In Group Loyalty (Violato, 2020). Further research is required to disentangle effects of profession and personality.

Cognitive load influenced PD, however, the results were counter to expectations that HCL would produce lower PD. Positive deviance occurred at a lower rate in the LCL condition for RT students, but not the ACP students. Though unexpected, when considered alongside the differences in approach and behaviour of the RT and ACP students the result is interpretable. Generally, RT students appeared to have a greater desire to avoid conflict and were not as direct in their approach to the doctor as the ACP students. The RT students took more time before engaging with the doctor and were more distant physically. In the HCL scenario, the distressed family member appeared to bring the students attention to the rapid desaturation that was occurring and the urgency of the situation. Conversely, in the LCL scenario RT students appeared to take longer to notice the sats and realize the danger the patient was in. Without the urgency created by the distressed family member participants may have been less likely to engage in PD with the desire to avoid conflict being stronger than the concern for the patient’s condition, leading to greater obedience.

For some participants there may be an effect of the Yerkes-Dodson law (Yerkes & Dodson, 1908) on PD. A certain level of arousal, whether physiological, cognitive, or emotional may be necessary to induce people to speak up. Determining differences in arousal thresholds for PD could be valuable in predicting, and explaining why, certain people did not engage in PD in the LCL condition or at what degree of patient danger a person will speak up.

The three-way interaction of Cognitive Load x Program x Positive Deviance was non-significant; however, the z score was comparable to the effect of Cognitive Load and Program separately and the p value neared 0.05. Higher order effects generally require a larger sample size for detection (Hong et al., 2020), a three-way interaction would likely have become significant with a slightly larger sample.

The Writing Task did not influence the rate of PD. Though the method was not successful in the present study, the strong existing evidence for the underlying cognitive mechanisms (Blanton & Christie, 2003; Cialdini, 2006; Holmes, et al., 2014; Wojcieszak, 2011) indicate that the approach still holds promise. The cause for the lack of success of the writing task is uncertain, but a brief writing task alone may not be strong enough to elicit the change in self-concept necessary to alter behavior. Further, as the true purpose of the task was not made apparent to students it may have lacked the necessary salience to be impactful. It is possible that a more involved activity such as a research report or presentation on compliance in healthcare that is integrated in a curriculum component related to speaking up and challenging authority may be more effectual.

### Secondary analysis

Several individual characteristics had some predictive value for the likelihood of engaging in PD. Despite suboptimal performance of the model selected, the classification accuracy was only 10% better than baseline accuracy and the Kappa values were low, the purpose was not to perfectly predict who would engage in PD. Rather several variables were identified for further investigation.

Ethnicity appears to play some role in obedience aligning with predictions of MF and cross-cultural theories (Graham et al., 2018). Three of the MFQ subscales were predictive of PD. Most notably In Group Loyalty (IGL) was a negative predictor of PD. Participants high in IGL may more strongly identify with the team and would perceive speaking up as being disloyal to the group. In the same sample, Violato (2020), showed cultural/ethnic background was a predictor of IGL. Possible cultural/ethnic behavioural differences may be manifested, in part, as a result of MF. Interestingly, Respect for Authority (RFA) had no predictive value. Overall, the sample scored low on RFA (Graham et al., 2008), which generally is not a very strong Western cultural value (Haidt, 2012). If the study were conducted with a sample from a different cultural context, RFA may appear as a negative predictor of PD. Future research, with a larger sample size, including more professions and focused sampling to include proportional levels of various cultural/ethnic backgrounds could provide further insight to the influence of MF. Due to the small sample size the results related to ethnicity should be interpreted as inferences, and direct conclusions should not be drawn.

Confidence in airway management was found to be a negative predictor of PD. Participants that score themselves higher in airway management confidence may be overconfident, and those less confident will have heightened attentiveness to the urgency of the situation because they believe they are less capable of managing the situation themselves, or are more attune to the danger, a possible Dunning-Kruger effect (Those lower in ability tend to be over-confident in their abilities) (Dunning, 2011).

Due to the non-uniform use of the responsibility phrase it was not possible to determine the specific extent of the effect of displacement of responsibility. Still, it does appear displacement of responsibility was influential in inhibiting PD. All participants that did not engage in PD heard the phrase and the observational analysis showed the use of the phrase was highly influential with some participants. The present findings along with previous findings (Bould et al., 2015; Friedman et al., 2015; Violato, King, & Bulut, 2020) indicate displacement of responsibility is an important variable for future study.

### Observations

The observational data informed the interpretation of the results of the primary and secondary research questions and led to further insights. In meta-analyses by Griesdale et al (2012) and Su et al (2011) the time for intubation with experts using a direct laryngoscope in a normal airway ranged from 13–66.7 and 17–93 s, respectively. In the present study the length of time to PD on average was 77 s, falling within the range identified by Su et al (2011). When considering the numerous intubation attempts, de-saturation of the patient, and the dismissiveness of the doctor towards the participant those that engage in PD, at the sample level, appear to have done so within a reasonable time frame. However, the distributions indicate a large amount of variability in individual performance with four different behaviour types: Direct-action, Delayed action/Realization, Inaction, and Frustrated Inaction. Interestingly, it was mentioned to students in the pre-brief before entering the scenario that they were needed “right away” and the situation was urgent. Despite the urgency of the situation there may have been uncertainty as to how to proceed, what amount of help or involvement should be offered, or a degree of fear about doing something. It is also possible that the nature of simulation reduced the perceived urgency.

One of the most interesting aspects of the observational analysis was the physical agitation displayed by many participants, both RT and ACP students. Physical agitation has been previously observed in compliance studies (Asch, 1951; Milgram, 1974) as an outward expression of the difficulty of breaking with the powerful implicit cognitive structures and social norms that create obedience and conformity. The physical agitation of participants is a visible example of the incredibly strong inherent forces of compliance. Reaching the point of physical demonstrations of frustration show how it is easy to ask questions or make suggestions but moving from “*I think we should bag the patient*” to “*Do you mind taking that laryngoscope out of that patient please*” can be extremely difficult. The effect was further emphasized by participants laughing or other expressions of relief after the scenario.

### Limitations

There were four primary limitations to the study. (1) The ability and confidence of the SPs playing the physicians. The SP’s were challenged to make the procedure appear realistic in the first simulations. The actors were also inconsistent with the responsibility phrase and occasionally delivered it at the wrong time or multiple times. As noted by the facilitators the actors tended to act dismissively towards the participants rather than authoritatively or aggressively. The actors had been instructed to act in an authoritative manner, and given examples, however, the novelty of the role and performing the laryngoscopy may have diminished this. Future studies should provide more training to the actors or use health professionals unknown to students and experienced in the procedure being used.

(2) Discrepancies in the perceived authenticity of the simulation. Aspects of the study that appeared to be inauthentic to students, such as the doctors lack of urgency was not echoed by the facilitators. Facilitators thought the doctor could have been more aggressive, forceful, and emotionally intense to increase the discomfort and challenge of the scenario. The facilitators wondered if the low aggression made it easier for the students to challenge when they otherwise would not have. That said, facilitators noted that what students saw as lack of urgency may have been interpreted by a more experienced practitioner as extreme calmness from an experienced anesthesiologist. One student mentioned that the doctor appeared somewhat “disheveled” in their appearance, and this seemed inauthentic, however, a facilitator noted that it was not uncommon to encounter doctors with a “disheveled” appearance during an airway emergency.

Interestingly, the quality of the SPs reinforces the strength of the influence of authority and hierarchies. Despite SP’s struggles there were participants that did not engage in PD even when the procedure was being performed very incorrectly, e.g. participants #88821 and #100001. The doctor, though generally appearing incompetent, still affected the participants ability to speak up. A final limitation with authenticity was that CL was not directly measured and a quantitative difference between conditions cannot be determined; as a result, it cannot be conclusively stated the family member condition provided authentically HCL.

(3) Being a simulation possibly diminished how seriously participants took the situation. Despite the simulation setting it appeared participants took the scenario seriously, no participants acted aloof or did not engage with the situation. Additionally, the aural and visual frustration and agitation demonstrated by participants and relieved laughing and statements after the simulation indicates that the scenario was sufficiently engaging and difficult, evoking a psychological and emotional response.

(4) The timing of the study and delivery of the writing task. In the weeks prior to the writing task students had received education on speaking up. The potential effect of the writing task may have been washed out by this prior education. Had the writing task been delivered and the study conducted prior to instruction on speaking up the writing task may have influenced behaviour. Conversely, the limitation of timing of the study was also a strength. Students had received training on speaking up only a couple of weeks prior to the simulation, yet 36% did not engage in PD. As one facilitator noted, “*We just went over this stuff two weeks ago. They should have all spoken up*”. The power of a situation can strongly influence individuals (Lankford, 2009; Zimbardo, 2011) even after recent education and training directly related to the situation.

### Implications

#### Education

The identification of four general types of behaviour during the compliance scenario indicates that it is important to consider individual variability when developing interventions for PD or speaking up. It is likely instructors need to be more intentional about teaching strategies to account for underlying individual differences. This could include teaching simple and explicit rule-based strategies for speaking up.

The difficulty students had in speaking up after having received instruction two weeks prior points to a need to move beyond simple didactic training in speaking up to something that can be more influential and longer lasting. This was attempted with the Writing Task in the present study, and though it did not demonstrate an effect, the approach merits further study. Simultaneously, the facilitators thought the simulation was interesting, strongly supplemented prior instruction and was a positive learning experience for the students *“To see the student’s reactions and the positive parts of the experience that they took from it was valuable.” * Prior experience with obedience and patient risk is likely important for speaking up. Simulation training specifically designed to address PD and speaking up, such as the scenario in the present study, in conjunction with robust and comprehensive debriefing could be valuable for preparing students to engage in PD in clinical settings.

Ethnic/cultural background is also likely an important variable to understand for compliance and education. Understanding differences related to ethnic/cultural backgrounds is especially important as countries, like Canada, become more diverse (Government of Canada, 2020), the number of different healthcare roles grow e.g. Health Care Aides and Practical Nurses (Kilpatrick et al., 2020), and people from diverse backgrounds enter more health professional roles. It may be particularly important for those with prior experience in healthcare in parts of the world where the manner of practice is less patient centered and more authoritarian or paternalistic (Triscott et al., 2016). Due to cultural differences previously experienced health professionals may need specific education on the importance and acceptability of PD.

#### Research

The present study helps to demonstrate the ecological validity and generalizability of using simulated scenarios for testing non-technical skills training. Ecological validity is supported by real world cases, such as that of Elaine Bromiley (Harmer, 2005) where a physician, or physicians, demonstrate fixation or resistance to the exclusion of external inputs or warnings, whether instrumentational (blood oxygen saturation) or human (a colleague’s statement of concern). Simulation is also an advantageous setting for conducting experiments that would not be possible in a naturalistic setting. The high degree of engagement and elicitation of an emotional reaction from participants indicates that a simulation scenario can provide adequate clinical verisimilitude for testing interpersonal interactions. Experiments are particularly important for the interprofessional field. It is necessary to not simply transpose findings or analogies from other fields (Breitbach et al., 2017) or infer likely effects, but to test hypotheses (Smets, 2018).

Simulation is also advantageous for psychological experiments where concerns are often raised about context and the generalizability and applicability of the results in a “real world” scenario (Durgin et al., 2012). Simulation can help address the critique of the lack of real-world validity or verisimilitude leveled at psychological experiments (Bless & Burger, 2016) and performing high impact experiments is a necessity (Benjamin & Simpson, 2009).

Continued investigation of internal mediating mechanisms and individual differences is necessary. Further research on personality traits will be important for understanding and predicting PD and obedience. Experience, including professional and life experience, and self-confidence and self-efficacy should also be investigated further. To better understand patient safety in the context of IPECP and the effects of individual differences in experience, education, and training it is necessary to go beyond system approaches. There is a broad spectrum of health professions and levels of training from students to experts, and variable education on speaking up. Expanding research on compliance and PD to all health professions and levels of training will provide greater insight into team functioning and professional and educational differences. Incorporating psychological theory in research will assist in understanding how individuals behave and how teams influence individual behaviour. Taken together, studying the range of health professions, individuals, and group influences, it will be possible to understand not only how IPECP can produce harm but also how to leverage this knowledge to improve patient safety. Deeper understanding and insight to obedience in healthcare can be attained beyond the broad acknowledgment of student-practitioner hierarchies.

## Conclusion

The present study addressed important variables of obedience in an interprofessional setting helping to examine the complexity of compliance in healthcare. The physical and social environment are integrated and interact with the individual. Obedience to authority is a very powerful innate heuristic that can influence behaviour in interprofessional teams, including in unexpected ways through variables such as CL, hierarchical structure, displacement of responsibility, and individual differences. The present study demonstrated how LCL and displacement of responsibility can inhibit PD for some individuals. Several individual characteristics were found that are likely important for PD and obedience requiring further investigation. Evidence for the ecological validity of using simulation for studying compliance scenarios was also developed. It is important to continue to expand research and understanding around these variables to make informed changes to the individual and the social environment to reduce pressures that produce compliance, attempt to increase PD, and ultimately reduce harm to patients.

## Supplementary Information

Below is the link to the electronic supplementary material.

Supplementary file1 (DOCX 47 kb)

## References

[CR1] Alingh CW, Van Wijngaarden JDH, Van De Voorde K, Paauwe J, Huijsman R (2019). Speaking up about patient safety concerns: The influence of safety management approaches and climate on nurses’ willingness to speak up. BMJ Quality and Safety.

[CR2] Ariely D (2008). Predictably irrational: The hidden forces that shape our decisions.

[CR3] Asch SE, Guetzkow H (1951). Effects of group pressure upon the modification and distortion of judgments. Groups, leadership and men; research in human relations.

[CR4] Bainbridge L, Regehr G, Orchard CA, Bainbridge L (2015). Should there be an “I” in Team? A new perspective on developing and maintaining collaborative networks in health professional care. Interprofessional client-centered collaborative practice: What does it look like? How can it be acheived?.

[CR5] Baker, K. (2019). Conformity and Cognitive Load in an Asch-Like Paradigm Study. Unpublished Thesis. Concordia.

[CR6] Bandura A (1999). Moral disengagement in the perpetration of inhumanities. Personality and Social Psychology Review.

[CR7] Bandura A (2002). Selective moral disengagement in the exercise of moral agency. The Journal of Moral Education.

[CR8] Baron RS, Vandello JA, Brunsman B (1996). The forgotten variable in conformity research: Impact of task importance on social influence. Journal of Personality and Social Psychology.

[CR9] Barrett DW (2017). Social influence In the wiley-blackwell encyclopedia of social theory.

[CR10] Barzallo Salazar MJ, Minkoff H, Bayya J, Gillett B, Onoriode H, Weedon J, Altshuler L, Fisher N (2014). Influence of surgeon behavior on trainee willingness to speak up: A randomized controlled trial. Journal of the American College of Surgeons.

[CR11] Benjamin LT, Simpson JA (2009). The power of the situation: The impact of milgram’s obedience studies on personality and social psychology. American Psychologist.

[CR12] Beran T (2015). Research advances in conformity to peer pressure: A negative side effect of medical education. Health Professions Education.

[CR13] Beran T, Drefs M, Kaba A, Al Baz N, Al Harbi N (2015). Conformity of responses among graduate students in an online environment. Internet and Higher Education.

[CR14] Beran T, Kaba A, Caird J, McLaughlin K (2014). The good and bad of group conformity: A call for a new programme of research in medical education. Medical Education.

[CR15] Beran TN, McLaughlin K, Al Ansari A, Kassam A (2013). Conformity of behaviors among medical students: Impact on performance of knee arthrocentesis in simulation. Advances in Health Sciences Education.

[CR16] Blanton H, Christie C (2003). Deviance regulation: A theory of action and identity. Review of General Psychology.

[CR17] Blass T (1999). The milgram paradigm after 35 years: Some things we now know about obedience to authority’. Journal of Applied Psychology.

[CR18] Blenkinsopp J, Snowden N, Mannion R, Powell M, Davies H, Millar R, McHale J (2019). Whistleblowing over patient safety and care quality: A review of the literature. Journal of Health Organization and Management.

[CR19] Bless H, Burger AM (2016). A closer look at social psychologists’ silver bullet: Inevitable and evitable side effects of the experimental approach. Perspectives on Psychological Science.

[CR20] Bocchiaro P, Zimbardo P (2017). On the dynamics of disobedience: Experimental investigations of defying unjust authority. Psychology Research and Behavior Management.

[CR21] Bould MD, Sutherland S, Sydor DT, Naik V, Friedman Z (2015). Residents’ reluctance to challenge negative hierarchy in the operating room: A qualitative study. Canadian Journal of Anesthesia/journal Canadien D’anesthésie.

[CR22] Breitbach AP, Reeves S, Fletcher SN (2017). Health care as a team sport?-Studying athletics to improve interprofessional collaboration. Sports (Basel Switzerland).

[CR23] Calhoun A, Boone MC, Porter M, Miller K (2014). Using simulation to address hierarchy-related errors in medical practice. The Permanente Journal.

[CR24] Campitelli G, Gobet F (2010). Herbert Simon’s decision-making approach: Investigation of cognitive processes in experts. Review of General Psychology.

[CR25] Cialdini RB (2006). Influence: The psychology of persuasion.

[CR26] Cialdini RB, Goldstein NJ (2004). Social influence: Compliance and conformity. Annual Review of Psychology.

[CR27] Cialdini RB, Trost MR, Fiske ST, Lindzey G (1998). Social influence: Social norms, conformity, and compliance. The Handook of Social Psychology.

[CR28] Cohen JB, Patel SY (2020). Getting to zero patient harm: From improving our existing tools to embracing a new paradigm. Anesthesia and Analgesia.

[CR29] Cosby KS, Croskerry P, Cosby K, Graber ML, Singh H (2017). Do teams make better diagnoses. Diagnosis: Interpreting the shadows.

[CR30] Croskerry P, Cosby KS, Graber ML, Singh H (2017). Diagnosis: Interperting the shadows.

[CR31] Daly Guris RJ, Duarte SS, Miller CR, Schiavi A, Toy S (2019). Training novice anaesthesiology trainees to speak up for patient safety. British Journal of Anaesthesia.

[CR32] Del Giudice M (2009). On the real magnitude of psychological sex differences. Evolutionary Psychology.

[CR33] Delaloye, N. J. (2017). *An Exploration of Deference Behaviours Exhibited within the Paediatric Resuscitation Environment* [University of Calgary]. 10.1017/CBO9781107415324.004

[CR34] Delaloye NJ, Tobler K, O’Neill T, Kotsakis A, Cooper J, Bank I, Gilfoyle E (2017). Errors during resuscitation: The impact of perceived authority on delivery of care. Journal of Patient Safety.

[CR35] Didier A, Dzemaili S, Perrenoud B, Campbell J, Gachoud D, Serex M, Staffoni-Donadini L, Franco L, Benaroyo L, Maya ZS (2020). Patients’ perspectives on interprofessional collaboration between health care professionals during hospitalization: A qualitative systematic review. JBI Evidence Synthesis.

[CR36] Doğruyol B, Alper S, Yilmaz O (2019). The five-factor model of the moral foundations theory is stable across WEIRD and non-WEIRD cultures. Personality and Individual Differences.

[CR37] Dunning D, Olson JM, Zanna MP (2011). The Dunning-Kruger effect: On being ignorant of one's own ignorance. Advances in experimental social psychology.

[CR38] Durgin FH, Klein B, Spiegel A, Strawser CJ, Williams M (2012). The social psychology of perception experiments: Hills, backpacks, glucose, and the problem of generalizability. Journal of Experimental Psychology: Human Perception and Performance.

[CR39] Elfering A, Grebner S, Ebener C (2015). Workflow interruptions, cognitive failure and near-accidents in health care. Psychology, Health and Medicine.

[CR40] Faul F, Erdfelder E, Lang A-G, Buchner A (2007). G*Power 3: A flexible statistical power analysis program for the social, behavioral, and biomedical sciences. Behavior Research Methods.

[CR41] Festinger L (1957). A theory of cognitive dissonance.

[CR42] Festinger L, Carlsmith J (1959). Cognitive consequences of forced compliance. Journal of Abnormal and Social Psychology.

[CR43] Fino E, Di Campli S, Patrignani G, Mazzetti M (2019). The modulating role of gender and aggression in emotional reactions of nursing students: A cross-sectional study. Journal of Advanced Nursing.

[CR44] Frenk J, Chen L, Bhutta ZA, Cohen J, Crisp N, Evans T, Fineberg H, Garcia P, Ke Y, Kelley P, Kistnasamy B, Meleis A, Naylor D, Pablos-Mendez A, Reddy S, Scrimshaw S, Sepulveda J, Serwadda D, Zurayk H (2010). Health professionals for a new century: Transforming education to strengthen health systems in an interdependent world. The Lancet.

[CR45] Friedman J, Hastie T, Tibshirani R (2010). Regularization paths for generalized linear models via coordinate descent. Journal of Statistical Software.

[CR46] Friedman Z, Hayter MA, Everett TC, Matava CT, Noble LMK, Bould MD (2015). Power and conflict: The effect of a superior’s interpersonal behaviour on trainees’ ability to challenge authority during a simulated airway emergency. Anaesthesia.

[CR47] Friedrich J (1993). Primary error detection and minimization (PEDMIN) Strategies in social cognition: A reinterpretation of confirmation bias phenomena. Psychological Review.

[CR48] Garden AL, Weller JM (2017). Speaking up: Does anaesthetist gender influence teamwork and collaboration?. British Journal of Anaesthesia.

[CR49] Gastil J, Black L, Moscovitz K (2008). Ideology, attitude change, and deliberation in small face-to-face groups. Political Communication.

[CR50] Gigerenzer G (2010). Moral satisficing: Rethinking moral behavior as bounded rationality. Topics in Cognitive Science.

[CR51] Gigerenzer G, Goldstein DG (1996). Reasoning the fast and frugal way: Models of bounded rationality. Psychological Review.

[CR52] Gilbert J (2013). Interprofessional—education, learning, practice and care. Journal of Interprofessional Care.

[CR53] Government of Canada. (2020). *Immigration, Refugees and Citizenship Canada Departmental Plan 2020–2021*. https://www.canada.ca/en/immigration-refugees-citizenship/corporate/publications-manuals/departmental-plan-2020-2021/departmental-plan.html#core2

[CR54] Graham, J., Haidt, J., & Nosek, B. (2008). *The Moral Foundations Questionnaire (MFQ-30)*. www.MoralFoundations.org

[CR55] Graham J, Haidt J, Motyl M, Meindl P, Iskiwitch C, Gray K, Graham J (2018). Moral foundations theory: On the advantages of moral pluralism over moral monism. Atlas of moral psychology.

[CR56] Green B, Oeppen RS, Smith DW, Brennan PA (2017). Challenging hierarchy in healthcare teams – ways to flatten gradients to improve teamwork and patient care. British Journal of Oral and Maxillofacial Surgery.

[CR57] Greenwald AG, Bellezza FS, Banaji MR (1988). Is self-esteem a central ingredient of the self-concept?. Personality and Social Psychology Bulletin.

[CR58] Griesdale DEG, Liu D, McKinney J, Choi PT (2012). Glidescope ® video-laryngoscopy versus direct laryngoscopy for endotracheal intubation: A systematic review and meta-analysis. Canadian Journal of Anesthesia.

[CR59] Grzyb T, Doliński D, Trojanowski J, Bar-Tal Y (2018). Cognitive structuring and obedience toward authority. Personality and Individual Differences.

[CR60] Haidt J (2001). The emotional dog and its rational tail: A social intuitionist approach to moral judgment. Psychology Review.

[CR61] Haidt J (2012). The righteous mind: Why good people are divided by politics and relgion.

[CR62] Harmer, M. (2005). The Case of Elaine Bromiley: Independent Report on the death of Elaine Bromiley.

[CR63] CAE Healthcare. (2017). *CAE iStan*. https://caehealthcare.com/media/files/TechSheets/iStan-TechSheet.pdf

[CR64] Hémon B, Michinov E, Guy D, Mancheron P, Scipion A (2020). Speaking up about errors in routine clinical practice: A simulation-based intervention with nursing students. Clinical Simulation in Nursing.

[CR65] Hogarth R (2001). Educating intuition.

[CR66] Holmes CL, Harris IB, Schwartz AJ, Regehr G (2014). Harnessing the hidden curriculum: A four-step approach to developing and reinforcing reflective competencies in medical clinical clerkship. Advances in Health Sciences Education.

[CR67] Hong M, Jacobucci R, Lubke G (2020). Deductive data mining. Psychological Methods.

[CR68] Howell D (2010). Log-linear analysis In statistical methods for psychology.

[CR69] Islam G, Zyphur MJ (2005). Power, voice, and hierarchy: Exploring the antecedents of speaking up in groups. Group Dynamics.

[CR70] James G, Witten D, Hastie T, Tibishirani R (2013). Linear model selection and regularization In an introduction to statistical learning.

[CR71] Jamovi. (2020). *The jamovi project* (1.2). https://www.jamovi.org

[CR72] Kaba A, Beran TN (2016). Impact of peer pressure on accuracy of reporting vital signs: An interprofessional comparison between nursing and medical students. Journal of Interprofessional Care.

[CR73] Kaba A, Beran TN, White D (2016). Accuracy of interpreting vital signs in simulation: An empirical study of conformity between medical and nursing students. Journal of Interprofessional Education and Practice.

[CR74] Kaba A, Wishart I, Fraser K, Coderre S, Mclaughlin K (2016). Are we at risk of groupthink in our approach to teamwork interventions in health care?. Medical Education.

[CR75] Kilpatrick K, Paquette L, Jabbour M, Tchouaket E, Fernandez N, Al Hakim G, Landry V, Gauthier N, Beaulieu MD, Dubois CA (2020). Systematic review of the characteristics of brief team interventions to clarify roles and improve functioning in healthcare teams. PLoS ONE.

[CR76] Kohn, L. T., Corrigan, J. M., & Donaldson, M. S. (1999). To err is human: Building a safer health system. In *Institute of Medicine*. Institute of Medicine. 25077248

[CR77] Küfner ACP, Back MD, Nestler S, Egloff B (2010). Tell me a story and I will tell you who you are! Lens model analyses of personality and creative writing. Journal of Research in Personality.

[CR78] Kuhn, M. (2019). *caret: Classification and Regression Training* (R package version 6.0–84). https://cran.r-project.org/package=caret

[CR79] Kuo SY, Wu JC, Chen HW, Chen CJ, Hu SH (2020). Comparison of the effects of simulation training and problem-based scenarios on the improvement of graduating nursing students to speak up about medication errors: A quasi-experimental study. Nurse Education Today.

[CR80] Langeron O, Bourgain JL, Francon D, Amour J, Baillard C, Bouroche G, Chollet Rivier M, Lenfant F, Plaud B, Schoettker P, Fletcher D, Velly L, Nouette-Gaulain K (2018). Difficult intubation and extubation in adult anaesthesia. Anaesthesia Critical Care and Pain Medicine.

[CR81] Lankford A (2009). Promoting aggression and violence at Abu Ghraib: The U.S. military’s transformation of ordinary people into torturers. Aggression and Violent Behavior.

[CR82] Lapkin S, Levett-Jones T, Gilligan C (2013). A systematic review of the effectiveness of interprofessional education in health professional programs. Nurse Education Today.

[CR83] Lewin K (1947). Frontiers in group dynamics. Human Relations.

[CR84] Lewin K (1947). Frontiers in group dynamics. Human Relations.

[CR85] Lönnqvist JE, Irlenbusch B, Walkowitz G (2014). Moral hypocrisy: Impression management or self-deception?. Journal of Experimental Social Psychology.

[CR86] Mak-van der Vossen M, Teherani A, van Mook WNKA, Croiset G, Kusurkar RA (2018). Investigating US medical students’ motivation to respond to lapses in professionalism. Medical Education.

[CR87] Martinez W, Lehmann LS, Thomas EJ, Etchegaray JM, Shelburne JT, Hickson GB, Brady DW, Schleyer AM, Best JA, May NB, Bell SK (2017). Speaking up about traditional and professionalism-related patient safety threats: A national survey of interns and residents. BMJ Quality and Safety.

[CR88] Matsuo A, Sasahara K, Taguchi Y, Karasawa M (2019). Development and validation of the Japanese moral foundations dictionary. PLoS ONE.

[CR89] McCutcheon LRM, Haines ST, Valaitis R, Sturpe DA, Russell G, Saleh AA, Clauson KA, Lee JK (2020). Impact of interprofessional primary care practice on patient outcomes: A scoping review. SAGE Open.

[CR90] Mcgrath A (2017). Dealing with dissonance: A review of cognitive dissonance reduction. Social and Personality Psychology Compass.

[CR91] Mikhail J, Vargas M, Doris J (2021). Moral intuitions and moral natavism. The oxford handbook of moral psychology.

[CR92] Milgram S (1974). Obedience to authority: An experimental view.

[CR93] Milligan F, Wareing M, Preston-Shoot M, Pappas Y, Randhawa G, Bhandol J (2017). Supporting nursing, midwifery and allied health professional students to raise concerns with the quality of care: A review of the research literature. Nurse Education Today.

[CR94] Nilsson A, Erlandsson A (2015). The moral foundations taxonomy: Structural validity and relation to political ideology in Sweden. Personality and Individual Differences.

[CR95] O’Donovan R, McAuliffe E (2020). A systematic review exploring the content and outcomes of interventions to improve psychological safety, speaking up and voice behaviour. BMC Health Services Research.

[CR96] Okuyama A, Wagner C, Bijnen B (2014). Speaking up for patient safety by hospital-based health care professionals: A literature review. BMC Health Services Research.

[CR97] Omura M, Maguire J, Levett-Jones T, Stone TE (2017). The effectiveness of assertiveness communication training programs for healthcare professionals and students: A systematic review. International Journal of Nursing Studies.

[CR98] Oner C, Fisher N, Atallah F, Son MA, Homel P, Mykhalchenko K, Minkoff H (2018). Simulation-based education to train learners to “speak up” in the clinical environment: Results of a randomized trial. Simulation in Healthcare.

[CR99] Paradis E, Whitehead CR (2018). Beyond the lamppost: A proposal for a fourth wave of education for collaboration. Academic Medicine : Journal of the Association of American Medical Colleges.

[CR100] Pattni N, Arzola C, Malavade A, Varmani S, Krimus L, Friedman Z (2019). Challenging authority and speaking up in the operating room environment: A narrative synthesis. British Journal of Anaesthesia.

[CR101] Pattni N, Bould MD, Hayter MA, McLuckie D, Noble LMK, Malavade A, Friedman Z (2017). Gender, power and leadership: the effect of a superior’s gender on respiratory therapists’ ability to challenge leadership during a life-threatening emergency. British Journal of Anaesthesia.

[CR102] Peadon R, Hurley J, Hutchinson M (2020). Hierarchy and medical error: Speaking up when witnessing an error. Safety Science.

[CR103] Pian-Smith MCM, Simon R, Minehart RD, Podraza M, Rudolph J, Walzer T, Raemer D (2009). Teaching residents the two-challenge rule: A simulation-based approach to improve education and patient safety. Simulation in Healthcare.

[CR104] Pyszczynski T, Solomon S, Greenberg J, Arndt J, Schimel J (2004). Why do people need self-esteem? A theoretical and empirical review. Psychological Bulletin.

[CR105] Qualtrics. (2019). Qualtrics, Provo, Utah, USA

[CR106] R Core Team. (2019). *R: A language and environment for statistical computing.* (3.6.1 (2019–07–05)). R Foundation for Statistical Computing. https://www.r-project.org/

[CR107] Reeves S, Palaganas J, Zierler B (2017). An updated synthesis of review evidence of interprofessional education. Journal of Allied Health.

[CR108] Richardot S (2014). “You know what to do with them”: The formulation of orders and engagement in war crimes. Aggression and Violent Behaviour.

[CR109] Roussin CJ, Larraz E, Jamieson K, Maestre JM (2018). Psychological safety, self-efficacy, and speaking up in interprofessional health care simulation. Clinical Simulation in Nursing.

[CR110] Schug CH (1954). A study of attitude change toward debate propositions among high school and college debaters. Communication Education.

[CR111] Schwappach D, Sendlhofer G, Kamolz LP, Köle W, Brunner G (2019). Speaking up culture of medical students within an academic teaching hospital: Need of faculty working in patient safety. PLoS ONE.

[CR112] Sewell JL, Santhosh L, O’Sullivan PS (2020). How do attending physicians describe cognitive overload among their workplace learners?. Medical Education.

[CR113] Shanks LC, Chiu SH, Zelko MI, Fleming E, Germano S (2020). Speaking up to authority in a simulated medication error scenario. Clinical Simulation in Nursing.

[CR114] Smets BK (2018). There is more to behavioral economics than biases and fallacies. Behavioural Scientist.

[CR115] Stelzl I (2000). What sample sizes are needed to get correct significance levels for log-linear models?-A Monte Carlo study using the SPSS-procedure Hiloglinear. In Methods of Psychological Research Online.

[CR116] Su YC, Chen CC, Lee YK, Lee JY, Lin KJ (2011). Comparison of video laryngoscopes with direct laryngoscopy for tracheal intubation: A meta-analysis of randomised trials. European Journal of Anaesthesiology.

[CR117] Sydor DT, Bould MD, Naik VN, Burjorjee J, Arzola C, Hayter M, Friedman Z (2013). Challenging authority during a life-threatening crisis: The effect of operating theatre hierarchy. British Journal of Anaesthesia.

[CR118] Szulewski A, Howes D, Van Merriënboer JJG, Sweller J (2021). From theory to practice: The application of cognitive load theory to the practice of medicine. Academic Medicine.

[CR119] Tasca GA (2020). What is group dynamics?. Group Dynamics: Theory, Research, and Practice.

[CR120] Tesi A, Pratto F, Pierro A, Aiello A, Pratto F (2020). Group dominance in hierarchy-attenuating and hierarchy-enhancing organizations: The role of social dominance orientation, need for cognitive closure, and power tactics in a person-environment (Mis)fit perspective. Group Dynamics: Theory, Research, and Practice.

[CR121] Triscott JAC, Szafran O, Waugh EH, Torti JMI, Barton M (2016). Cultural transition of international medical graduate residents into family practice in Canada. International Journal of Medical Education.

[CR122] Violato, E. (2020). A comparison of traditional and data mining approaches for exploratory data analysis on a psychological measure using a small sample size. Available at SSRN: http://ssrn.com/abstract=3908201.

[CR123] Violato E, King S, Bulut O (2020). A multi-method exploratory study of health professional students’ experiences with compliance behaviours. BMC Medical Education.

[CR124] Vuurberg G, Vos JAM, Christoph LH, de Vos R (2019). The effectiveness of interprofessional classroom-based education in medical curricula: A systematic review. Journal of Interprofessional Education and Practice.

[CR125] Weiss M, Kolbe M, Grote G, Dambach M, Marty A, Spahn DR, Grande B (2014). Agency and communion predict speaking up in acute care teams. Small Group Research.

[CR126] Wellmon R, Lefebvre K, Ferry D (2017). Effects of high-fidelity simulation on physical therapy and nursing students’ attitudes toward interprofessional learning and collaboration. The Journal of Nursing Education.

[CR127] Wojcieszak M (2011). Deliberation and attitude polarization. Journal of Communication.

[CR128] World Health Organization. (2010). *Framework for Action on Interprofessional Education and Collaborative Practice*. 21174039

[CR1000] Yerkes RM, Dodson JD (1908). The Relation of Strength of Stimulus to Rapidity of Habit Formation. J Comp Neurol Psychol.

[CR129] Zimbardo PG (2011). Lucifer effect.

[CR130] Zou, H., & Hastie, T. (2005). Regularization and Variable Selection via the Elastic Net. *Journal of the Royal Statistical Society. Series B (Statistical Methodology), 67*(2), 301–320. http://www.jstor.org/stable/3647580

