# Supplementary material for: A behavioural study of obedience in health professional students
Source: Adv Health Sci Educ Theory Pract. 2021 Nov 22;27(2):293–321. doi: 10.1007/s10459-021-10085-4 (PMC9117351; doi:10.1007/s10459-021-10085-4)
Supplement: Supplementary file 1 — Supplementary file1 (DOCX 47 kb) [file 10459_2021_10085_MOESM1_ESM.docx]

**Supplemental Material: Questionnaires and Procedures**

**What month were you born?**

- January
- February
- March
- April
- May
- June
- July
- August
- September
- October
- November
- December

Q1 Please indicate your biological sex

- Male
- Female
- Other

Q2 Please indicate your age

|  | 0 | 10 | 20 | 30 | 40 | 50 | 60 | 70 | 80 | 90 | 100 |
| --- | --- | --- | --- | --- | --- | --- | --- | --- | --- | --- | --- |

| Age | 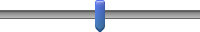 |
| --- | --- |

Q3 Please estimate your GPA

|  | 0 | 1 | 2 | 3 | 4 |
| --- | --- | --- | --- | --- | --- |

| GPA | 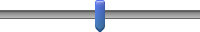 |
| --- | --- |

Q4 Please select the choice that best represents your ethnicity/geographic background

- Caucasian (European Origin)
- Hispanic (Latin America)
- South East Asian
- Chinese
- Japanese
- Pacific Islander
- African Canadian/American
- African
- India
- Middle East Origin
- Aboriginal
- Other
- Prefer not to indicate

Q5 Please estimate the number of times you have practiced in a simulated health care setting or with a simulator

_____________________________________________________________________________

Q19 Please indicate your level of experience with Airway Management from: *1 - Not at all Experienced* to *5 - Very Experienced*

|  | 1 | 2 | 3 | 4 | 5 |
| --- | --- | --- | --- | --- | --- |
| Experience |  |  |  |  |  |

Q20 Please indicate your level of confidence performing Airway Management: *1 - Not at all Confident* to *5 - Very Confident*

|  | 1 | 2 | 3 | 4 | 5 |
| --- | --- | --- | --- | --- | --- |
| Confidence |  |  |  |  |  |

Q40 Please estimate the number of hours of experience in the classroom, lab or simulation you have had with Airway Management

|  | 0 | 10 | 20 | 30 | 40 | 50 | 60 | 70 | 80 | 90 | 100 |
| --- | --- | --- | --- | --- | --- | --- | --- | --- | --- | --- | --- |

| Number of Hours | 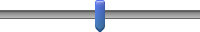 |
| --- | --- |

Q6 Please estimate the number of weeks of clinical experience you have had

|  | 0 | 25 | 50 | 75 | 100 |
| --- | --- | --- | --- | --- | --- |

| Number of Weeks | 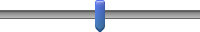 |
| --- | --- |

Q7 Please indicate your program

- Respiratory Therapy
- EMT – Paramedic

Q8 Please indicate your program year

|  | 0 | 1 | 2 | 3 |
| --- | --- | --- | --- | --- |

| Program Year | 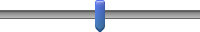 |
| --- | --- |

Q9 Counting all of your years of post-secondary education what year of post-secondary is this for you?

|  | 0 | 2 | 3 | 5 | 6 | 8 | 9 | 11 | 12 | 14 | 15 |
| --- | --- | --- | --- | --- | --- | --- | --- | --- | --- | --- | --- |

| Year of Post-Secondary | 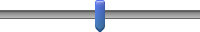 |
| --- | --- |

______________________________________________________________________________

For the next part of the questionnaire we will be asking you to complete a writing task. The purpose of the writing task is to provide data that will be used to complete a personality assessment as well as determine the validity of using writing tasks for assessing how different personality types learn in simulation. It has been shown that writing tasks can be used as an alternative and richer form of assessing personality traits by analyzing writing patterns (Kufner, Back, Nestler, & Egoff, 2010). You will be assigned to one of several different writing tasks, some of these tasks will be related to healthcare while others will be unrelated to healthcare. Assignment to the writing task is completely random. *(Kufner, A.C.P., Back, M.D., Nestler, S., & Egoff, B. (2010). Tell me a story and I will tell you who you are. Lens model analyses of personality and creative writing. Journal of Research in Personality, 44, 427-435.)*

**Educational Material Task**

Often in healthcare we are faced with situations where we are unsure how to act. Sometimes a person who is in a position of authority will ask us, or someone around us, to carry out a procedure that we think is incorrect. For example, a senior physician might ask us to deliver 50mg of a drug but we know this is incorrect and the proper dosage is 25mg, however we do not say anything and carry out the procedure as instructed. There are two parts to the writing task: **First**, please take time to write about how **NOT** speaking up when an authority figure acts incorrectly, or recommends action that is incorrect, can have negative outcomes for patients. Please be detailed and write about different negative outcomes, these may include situations you have experienced. **Second**, please write about a situation where an authority figure is asking you to carry out a task incorrectly and how you would act to ensure the best outcome for a patient. Please be detailed in describing how you would act. What words or phrases would you use? What would your body language be? What would you do if the authority figure was not accepting of your initial action?    


The more detail you can provide the better an understanding of your personality can be developed.

________________________________________________________________

**Neutral Task 1**

Writing about events in the past can be helpful in taking a reflective position that is useful in determining different personality traits. For this writing task we would like you to think back to the summer, specifically we would like you to think about the best summer vacation you can remember and write about that summer vacation in as much detail as possible.    

The more detail you can provide the better an understanding of your personality can be developed.

________________________________________________________________

**Neutral Task 2**

Becoming a healthcare professional requires a great deal of commitment and studying. For this writing task there two are parts:

First, in as much detail as possible please write about your favorite place to study. Where is that place? What does it look and smell like? Please feel free to go into as much detail as you like.

Second, please write about your study strategies. How do you like to study? What methods do you use? What time of day do you like to study? Please feel free to go into as much detail as you like. 
 
The more detail you can provide the better an understanding of your personality can be developed.

________________________________________________________________

**Neutral Task 3**

Writing about favorite events that we enjoy can be helpful in taking a reflective position that is useful in determining different personality traits. For this writing task we would like you to write about your favorite past-time outside of school. What is that activity and why do you enjoy it? Where do you do it? How frequently do you do it? Please feel free to go into as much detail as possible. 

The more detail you can provide the better an understanding of your personality can be developed.

________________________________________________________________

Thank you for completing the writing task. After you have completed the simulation you will be asked a few questions and may be asked to complete another brief writing task on a topic that you were NOT assigned to. To maintain the integrity of the personality assessment and your learning in the simulation we ask that you**PLEASE DO NOT DISCUSS** the writing task that you were assigned to with any of your peers participating in this study. Discussing the writing tasks with others can cause the second writing task you complete to not be effective and will not allow for an accurate measure of personality.
There is one more section of the questionnaire for you to complete.

-------------------------------------------------------------------------------------------------------------------------------The final section is a personality questionnaire. There are 32 questions. The results from the questionnaire will be used to compare and contrast with the writing task to help interpret the writing task and determine your personality type.

Part 1. When you decide whether something is **Right** or **Wrong**, to what extent are the following considerations relevant to your thinking? Please rate each statement.

|  | Not at all Relevant | Not Very Relevant | Slightly Relevant | Somewhat Relevant | Very Relevant | Extremely Relevant |
| --- | --- | --- | --- | --- | --- | --- |
| 1. Whether or not someone suffered emotionally |  |  |  |  |  |  |
| 2. Whether or not some people were treated differently than others |  |  |  |  |  |  |
| 3. Whether or not someone’s action showed love for his or her country |  |  |  |  |  |  |
| 4. Whether or not someone showed a lack of respect for authority |  |  |  |  |  |  |
| 5. Whether or not someone violated standards of purity and decency |  |  |  |  |  |  |
| 6. Whether or not someone was good at math |  |  |  |  |  |  |
| 7. Whether or not someone cared for someone weak or vulnerable |  |  |  |  |  |  |
| 8. Whether or not someone acted unfairly |  |  |  |  |  |  |
| 9. Whether or not someone did something to betray his or her group |  |  |  |  |  |  |
| 10. Whether or not someone conformed to the traditions of society |  |  |  |  |  |  |
| 11. Whether or not someone did something disgusting |  |  |  |  |  |  |
| 12. Whether or not someone was cruel |  |  |  |  |  |  |
| 13. Whether or not someone was denied his or her rights |  |  |  |  |  |  |
| 14. Whether or not someone showed a lack of loyalty |  |  |  |  |  |  |
| 15. Whether or not an action caused chaos or disorder |  |  |  |  |  |  |
| 16. Whether or not someone acted in a way that God would approve of |  |  |  |  |  |  |

|  | Strongly Disagree | Moderately Disagree | Slightly Disagree | Slightly Agree | Moderately Agree | Strongly Agree |
| --- | --- | --- | --- | --- | --- | --- |
| 17. Compassion for those who are suffering is the most crucial virtue |  |  |  |  |  |  |
| Part 2: Please read the following sentences and indicate your **Agreement** or **Disagreement** | | | | | | |
| 18. When the government makes laws, the number one principle should be ensuring that everyone is treated fairly |  |  |  |  |  |  |
| 19. I am proud of my country’s history |  |  |  |  |  |  |
| 20. Respect for authority is something all children need to learn |  |  |  |  |  |  |
| 21. People should not do things that are disgusting, even if no one is harmed |  |  |  |  |  |  |
| 22. It is better to do good than to do bad |  |  |  |  |  |  |
| 23. One of the worst things a person could do is hurt a defenseless animal |  |  |  |  |  |  |
| 24. Justice is the most important requirement for a society |  |  |  |  |  |  |
| 25. People should be loyal to their family members, even when they have done something wrong |  |  |  |  |  |  |
| 26. Men and women each have different roles to play in society |  |  |  |  |  |  |
| 27. I would call some acts wrong on the grounds that they are unnatural |  |  |  |  |  |  |
| 28. It can never be right to kill a human being |  |  |  |  |  |  |
| 29. I think it’s morally wrong that rich children inherit a lot of money while poor children inherit nothing |  |  |  |  |  |  |
| 30. It is more important to be a team player than to express oneself |  |  |  |  |  |  |
| 31. If I were a soldier and disagreed with my commanding officer’s orders, I would obey anyway because that is my duty |  |  |  |  |  |  |
| 32. Chastity is an important and valuable virtue |  |  |  |  |  |  |

### Compliance Simulation Procedures: for Facilitators

*Context:*

At the point of participating in the simulation the participants will have filled out a questionnaire that included demographic questions, a writing task and a personality questionnaire. This occurred approximately a week prior to the simulation.

The participants are under the impression that the study is a personality study. The participants believe that the writing task was a personality assessment that will be related to their performance in the simulation. The true purpose of the writing task is to help participants engage in positive deviance (treatment condition).

For the simulation portion of the study participants will be exposed to a high or low cognitive load scenario where an anesthetist is attempting to secure a patient’s airway. The primary variable of interest is if the participant engages in positive deviance. The participant will have two opportunities to speak up, if they engage in positive deviance twice the scenario will end.

**Positive Deviance**: When a person speaks up or engages in action that is counter to the incorrect instructions or actions of a person in a position of authority; e.g. in the present simulation scenario if the participant tells the physician that what they are doing is incorrect or that they should stop this would count as positive deviance.

*Procedures:*

Facilitator should remain in the sim area separate from the participants for the entirety of the simulation session.

1. Have personal device ready and loaded to the questionnaire start page
2. Call a participant from the waiting room to the simulation room.
3. Give the participant the device and have the participant complete the ID and deception check question.
4. Retrieve the device from the participant when they are finished.
5. Enter the simulation room.
6. A participant ID will be presented to you. Communicate this to the Sim Facilitator.
7. High or Low cognitive load will be presented to you on the questionnaire, select the option presented, indicate to the actors which scenario (high or low cognitive load will occur).
8. Ensure actors are prepared. Return to the participant.
9. Tell the participant they may enter the simulation
10. Observe the simulation.
11. When the point for positive deviance arises if the participant is positively deviant select the appropriate option in the questionnaire.
12. After the simulation is terminated take the participant to the debriefing room.
13. Activate audio recording
14. State aloud the Participant ID presented to you.
15. Proceed through the remainder of the questions on the questionnaire.
16. After completing the questions complete the debriefing, including the deception check.
17. Answer the question for the deception check.
18. Thank the participant for participating and dismiss them, reminding the participant not to discuss the true nature of the study with anyone.
19. Complete the survey and refresh the survey on the device (will have to delete browser history) and repeat 1-16.

### Simulation Procedures

**School of Health and Life Sciences**

**Simulation Centre**

**Compliance Behaviour During an Airway Emergency**

**Creation Date: February 2019**

**Public Scenario Title**

Compliance Behaviour During an Airway Emergency

**Scenario Description**

In an urban emergency department an Anesthesiologist has had two intubation attempts without success. The RT and/or ACP is called in by the charge nurse to assist with capturing the airway and as they enter the room the anesthetist begins to attempt intubation for the third time.

There are two different groups of students for this simulation research project, a high cognitive load group and a low cognitive load group.

For the high cognitive load group there will also be a standardized patient playing the role of a distraught family member who is asking lots of questions and saying things like “help my husband”.

**Scenario Objectives**

1. Ensure closed-loop communication between team members
2. Use NOD (name, occupation, duty) when first introducing self to patients and family members
3. Rapid Patient Assessment
4. Appropriately challenging inappropriate decision making

**Equipment and Supplies**

| Mannequin laying on bed, wearing hospital gown. | Sim cart |
| --- | --- |
| OPA’s | Suction set up with yankeur suction attached |
| Intubation Tray | Bougie Stylette |
| Bagger and mask |  |

**Scenario Flow**

Briefly describe progressive scenes (aka states or stages)

1^st^ Scenario:

1. Patient in hospital gown, laying on stretcher being bagged by an SP playing the role of the Anesthetist.
2. Mannequin Settings: RR 0 HR 118 BP 110/70 SpO2 88%
3. Students should introduce themselves and perform a rapid situation assessment/get a situation report from the Anesthetist.
4. As the student assesses the patient the anesthetist rushes into a third intubation attempt.
   1. As soon as the anesthetist inserts the laryngoscope in the mouth set the SpO2 to decrease to 65% over the next 60 seconds.
5. The student should challenge the anesthetist and suggest methods for increasing the patient’s SpO2 (ex. bagging with an OPA) and optimizing conditions for successfully capturing the airway (ex. Shoulder roll, sniffing position, difficult airway device)
   1. If/when the student challenges the anesthetist the anesthetist proceeds as-is with the intubation attempt anyways.
   2. If/when the student fails to challenge the anesthetist the HR is set to decrease to 40 over the next 60 seconds.
6. The student is provided with one more opportunity to challenge the Anesthetist.
   1. If/when the student challenges the anesthetist the anesthetist states “It’s ultimately my responsibility for what happens here, I need to get this tube in.”
      1. If the student challenges again the scenario concludes, and if they do not challenge again the scenario also concludes.
   2. If/when the student does not challenge the anesthetist the scenario concludes.

**Briefing (or Prebriefing) Information**

**Participant(s) in the Hot Seat**

You are working a shift at the UofA emergency department and have just returned from Lunch break when the charge nurse rushes over to you and says “we need you right away in room one, I think Dr. Anderson from Anesthesia is having trouble getting an airway on a sepsis patient that just arrived.

**Standardized Patient Information**

**One standardized patient will play the role of an anesthetist.**

The SP should be a Caucasian male between the age of 40 and 55, and of medium build (i.e. taller than 5’7 but shorter than 6’ if possible

The SP will have an earpiece mic so that the simulation facilitator can help guide their actions.

When the scenario begins the SP will be in the simulation room with intubation equipment open/used at the bedside. They will be ventilating the patient with a bagger and looking somewhat stressed out.

When the student enters the room and introduces themselves the SP will say in a rushed/hurried manner “I’m Dr. Anderson from anesthesia, I’m just about to give this intubation another try here.”

The SP will then begin to attempt intubation on the mannequin.

Further dialogue and actions will be guided through the earpiece by the simulation facilitator.

****High Cognitive Load Group Only (half of the students)** A second SP will play the role of the patient’s wife.**

This SP should be female between the age of 40 and 55. They will be at the patient’s bedside and be nervous/worried/distraught. Once the student enters the room and has an initial dialogue with the anesthetist the SP begins saying:

“What is going on?”

“Should all of those alarms be ringing?”

“What are you doing?”

“What do all those numbers mean?”

Further actions and dialogue will be guided by the simulation facilitator through an earpiece mic.

**Debriefing Information**

Debriefing will be performed as per the debrief protocol submitted to the NAIT research ethics board.

**Roles of the Facilitator through the Simulation Experience**

- Respect for learner opinions and psychological safety
- Belief in integrity of learning through simulation
- Manages upset/monopolizing/outlier individuals
